# Prospective analysis of the use of OnabotulinumtoxinA (BOTOX) in the treatment of chronic migraine; real-life data in 254 patients from Hull, UK

**DOI:** 10.1186/1129-2377-15-54

**Published:** 2014-09-01

**Authors:** Modar Khalil, Hassan W Zafar, Victoria Quarshie, Fayyaz Ahmed

**Affiliations:** 1 Specialist Registrar in Neurology, Hull Royal Infirmary, Anlaby Road, Hull HU3 2JZ, UK; 2 Specialist Headache Nurse, Hull Royal Infirmary, Anlaby Road, Hull HU3 2JZ, UK; 3 Consultant Neurologist, Hull Royal Infirmary, Anlaby Road, Hull HU3 2JZ, UK

## Abstract

**Background:**

Chronic migraine affects 2% of the population. It results in substantial disability and reduced quality of life. Medications used for prophylaxis in episodic migraine may also work in chronic migraine. The efficacy and safety of OnabotulinumtoxinA (BOTOX) in adults with chronic migraine was confirmed in the PREEMPT programme. However, there are few real-life data of its use.

**Method:**

254 adults with chronic migraine were injected with OnabotulinumtoxinA BOTOX as per PREEMPT Protocol between July 2010 and May 2013, their headache data were collected using the Hull headache diary and analysed to look for headache, migraine days decrements, crystal clear days increment in the month post treatment, we looked at the 50% responder rate as well.

**Results:**

Our prospective analysis shows that OnabotulinumtoxinA, significantly, reduced the number of headache and migraine days, and increased the number of headache free days. OnabotulinumtoxinA Botox also improved patients’ quality of life. We believe that these results represent the largest post-marketing cohort of patients treated with OnabotulinumtoxinA in the real-life clinical setting.

**Conclusion:**

OnabotulinumtoxinA is a valuable addition to current treatment options in patients with chronic migraine. Our results support findings of PREEMPT study in a large cohort of patients, we believe, is representative of the patients seen in an average tertiary headache centre. While it can be used as a first line prophylaxis its cost may restrict its use to more refractory patients who failed three oral preventive treatments.

## Background

Chronic migraine, defined as headaches on ≥15 days per month for ≥3 months, of which ≥8 days meet criteria for migraine without aura or respond to migraine-specific treatment [1,2], is estimated to affect 2% of the population [3,4]. It results in substantial disability and reduced quality of life (QoL) [5-7] and leads to an increased risk of anxiety and depression [8].

Chronic migraine has significant health, economic and social consequences [2,4,9-13]; patients with chronic migraine are more likely to use healthcare resources than those with episodic migraine (defined as migraine and <15 headache days per month) [2], and one in five chronic migraine sufferers cannot work due to the effect of the condition on their ability to lead a productive life [14]. Chronic migraine sufferers are also significantly more likely to report depression, anxiety, chronic pain and respiratory disorders than non-chronic migraine sufferers [13].

Medications used for prophylaxis in episodic migraine may also work in chronic migraine, although only topiramate has established evidence [15,16]. However, this and other unlicensed oral agents have limitations due to poor tolerability and/or adverse effects, and a considerable number of patients do not respond [1,17,18]. More invasive and costly options include greater occipital nerve block (invasive) and occipital nerve stimulation (costly) that have their own limitations and disadvantages to patients and the health service [19]. Chronic migraine management is further complicated by analgesic overuse [6,20-23]. Observational and clinical trials have shown that 50-80% of patients with chronic migraine overuse acute medication [24]. As to whether the two are separate entities or a complication of one another remains uncertain [25]. For patients who fail on oral therapies, there is also now the option of treatment with OnabotulinumtoxinA before resorting to these invasive and expensive options.

The efficacy and safety of OnabotulinumtoxinA in adults with chronic migraine was shown in the phase III Research Evaluating Migraine Prophylaxis Therapy (PREEMPT) clinical programme [18,26-29]. These data led to the licensing authorities granting approval for this toxin in chronic migraine. OnabotulinumtoxinA has also more recently been shown to result in clinically meaningful reductions in headache impact and improvements in Health-Related Quality of Life (HRQoL) [30]. Furthermore, recent long-term data have confirmed that most chronic migraine patients who initially respond to OnabotulinumtoxinA will maintain the response over at least two years, and a substantial minority will be able to discontinue treatment and do well without prophylactic therapy. However, some patients showed reduced response on repeated injections [31].

Despite being the only drug licensed for prophylaxis in chronic migraine [28,32], few patients are being offered OnabotulinumtoxinA due to widespread funding restrictions and few data exist in the real-life setting. The aim of this study was to examine the change in the frequency of migraine symptoms before and after treatment in the real-life setting.

## Methods

The data was collected in a public sector clinic in the United Kingdom where patients were treated free of charge on the National Health Service (NHS) under the guidance of National Institute of Clinical Excellence (NICE); the organisation often regarded as a watchdog to determine the cost-effectiveness of a treatment before recommending it on the NHS. The funding implications faced in other countries may be different, although the authors feel that the NICE recommendations may have impact in some other countries.

### Study participants

Adult patients with chronic migraine (defined according to the 2004 International Headache Society Criteria) [2] attending the Hull Migraine Clinic between the first of July 2010 and the 31st of May 2013 were offered OnabotulinumtoxinA after discussion of all available treatment options, depending on the treatments that they had already received. The Hull Migraine Clinic (Hull Royal Infirmary and Spire Hospital Hull and East Riding) is a tertiary headache centre that sees 1,200 new headache referrals each year from across the North of England. Patients seen towards the start of the study period were either approved through an Individual Funding Request (IFR) or volunteered for the Allergan sponsored training sessions. A number of these patients had only tried a single preventive treatment. However, following publication of National Institute for Health and Clinical Excellence (NICE) guidance in June 2012 [33], subsequent patients included were treated within the National Health Service (NHS) and were only given OnabotulinumtoxinA treatment after having failed at least three preventive treatments as per NICE guidance. All patients received free treatment at the point of entry. Patients had to consent to OnabotulinumtoxinA treatment according to the PREEMPT study protocol [29]. There was no randomisation in this prospective analysis; subjects were selected according to their clinical need if they had chronic migraine that was not satisfactorily managed by their current therapy. Of note, patients who fulfilled the criteria for medication overuse were not excluded since they represent patients in the real-life setting. Due to the high prevalence of medication overuse in chronic migraine, the IHS has allowed these subjects to be included in their guidelines for chronic migraine trials provided that they are stratified accordingly [34]. According to expert opinion, inclusion of medication overuse patients should be allowed within the classification of chronic migraine to accurately reflect the patient population seen in actual clinical practice [1].

### Study design

**Figure 1:**
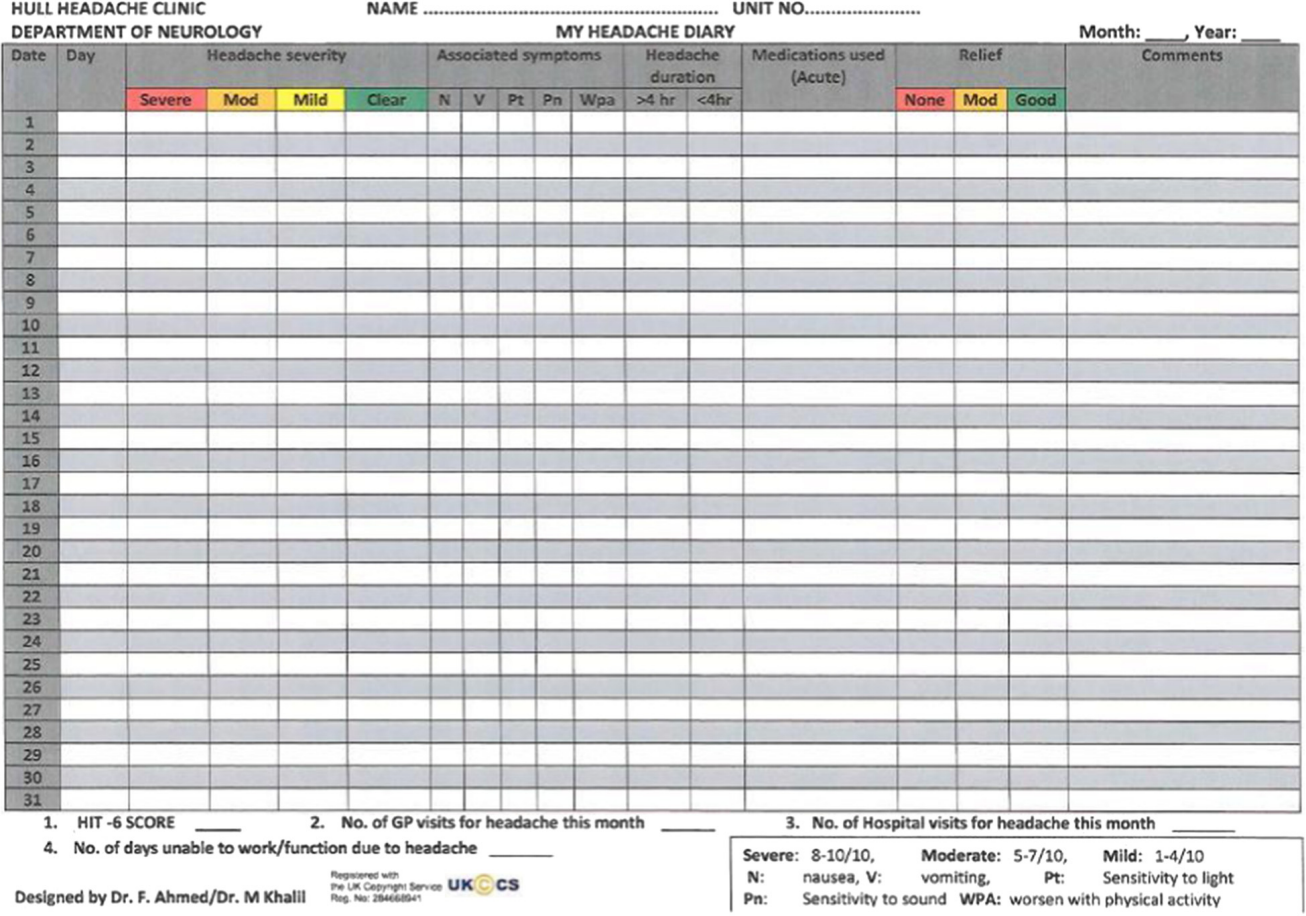
Hull headache diary.

Subjects were injected intramuscularly with OnabotulinumtoxinA according to the PREEMPT protocol, i.e. 155 units injected into 31 injection sites around the head and neck [29]. The paradigm includes follow the pain injections of up to further 45 units, although none of our patients received additional injections. Patients were asked to maintain a headache diary for at least 30 days prior to and continuously after receiving OnabotulinumtoxinA treatment. The Hull Headache Diary (shown below) (Figure 1) was used to capture data on headache [35]. The continuous diary filling was mandatory to assess response to treatment in order to determine whether patients were offered a repeat treatment.

### Study measures

From the completed patient diaries, assessments were made of headache days, migraine days and headache-free days; also, of analgesic medication use, triptan use, adverse events and days off work (if applicable). Quality of life was also measured through the Headache Impact Test (HIT-6) in patients receiving injections after the NICE guidance was published. For purpose of repeat treatment we used the responder criteria defined by NICE i.e. at least 30% reduction in headache days. As some patients showed marked reduction in migraine days than headache days we devised our own responder criteria (Hull Criteria) for analysing prospective patients in this study. A responder was defined as one with a 50% reduction in headache or migraine days, or an increment in headache free days twice that of the baseline in a 30-day period. Those with less than three headache free days were only classed as a responder if they achieved a minimum of six headache free days after the treatment. Some patients who failed all three parameters could still receive another OnabotulinumtoxinA injection if they perceived that the first injection had improved their QoL based on the patient’s perception or improvement on at least six points on the HIT-6 score, although as they did not fulfil the NICE responder criteria, further funding applications were made on exceptional grounds. A 50% and 75% response for each of the parameters, and those fulfilling two or all the three above parameters, were also analysed.

### Statistical analysis

The primary aim of the analysis was to compare the difference between outcome measurements made before and after treatment. All outcomes were measured on a continuous scale.

A statistical examination of the distribution of these outcomes found that they were skewed in their distribution for each set of measurements, and in terms of the change in values from pre- to post-treatment. As a result of these skewed distributions, the Wilcoxon matched-pairs test was used to compare the change in values over time.

For each patient, it was calculated whether they were a ‘responder’ based on either a 50% or 75% reduction in the number of days with symptoms. The exception was for headache free days where a responder was defined by either a two- or three-fold increase in the number of crystal clear days, provided there were at least three headache free days prior to treatment. P- Values of less than 0.05 were regarded as evidence of a statistically significant result.

HIT-6 was used to quantify the change in QoL. The HIT-6 score was analysed on a continuous scale, and an examination of the change in values over time indicated that the changes were normally distributed. As a result, the paired t-test was used to compare theHIT-6 values on the two occasions.

## Results

### Demographic and baseline headache characteristics

A total of 455 treatment cycles were given in all; of 284 patients injected, full data were available on 254 patients (55 male, mean age 48.6 years; range 19–77 years, 199 female (78% of cohort), mean age 44.06 years, range 19–91 years). Patients had the diagnosis of chronic migraine for a mean of 1.4 years (range ten months to three years) and reported daily headaches for a mean of 8.8 years (range 18 months to 30 years).

### Prior prophylactic treatments

Of the 254 patients, 240 (94.4%) had received (and failed due to lack of efficacy or intolerable side effects) three or more preventative treatments prior to OnabotulinumtoxinA; twelve patients (4.7%) had received two preventative drugs, and two patients (0.7%) had received one preventative treatment and opted by choice for OnabotulinumtoxinA treatment. Note that patients who received OnabotulinumtoxinA following failure to respond to one or two preventative treatments were given the treatment before NICE guidance was published.

### Acute analgesics overuse

Full data on acute analgesic use were available on 242 patients, of whom 122 (50.4%) fulfilled the criteria for misusing painkillers and/or triptans as per The International Classification of Headache Disorders [2] definition.

### Efficacy results

**Figure 2:**
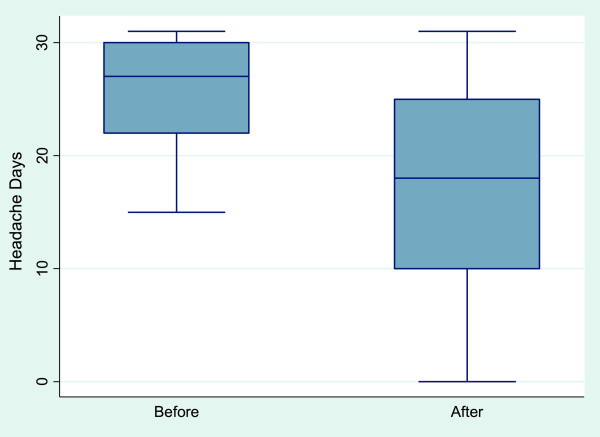
Change in headache days pre- and post-BOTOX in chronic migraine sufferers*. *In this box plot, the middle line is the median. The ‘box’ part represents the inter-quartile range (IQR), i.e. the middle half of the data. The ‘whiskers’ (i.e. the lines that come out from the box) then typically represent the minimum to maximum points. The exception is for points that are more than 1.5 times the IQR away from the box, in which case these are plotted separately. The value of 1.5 IQRs is chosen by convention in statistics.

**Figure 3:**
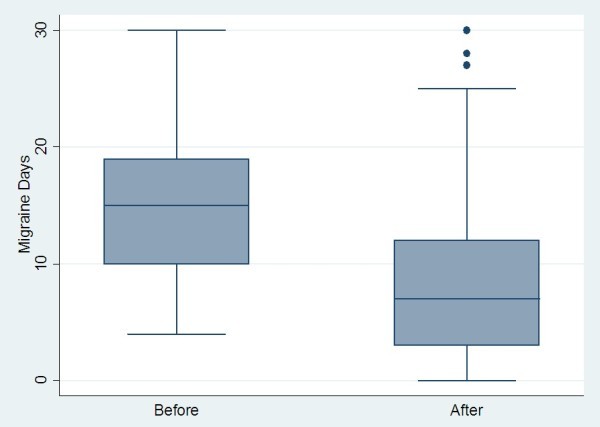
Change in migraine days pre- and post-BOTOX in chronic migraine sufferers.

**Figure 4:**
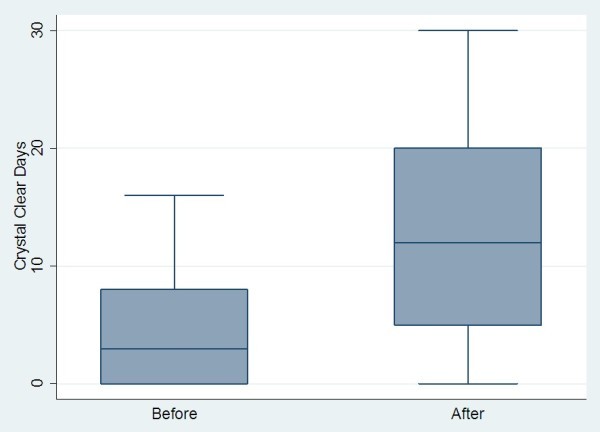
Change in crystal-clear days pre- and post-BOTOX in chronic migraine sufferers.

**Figure 5:**
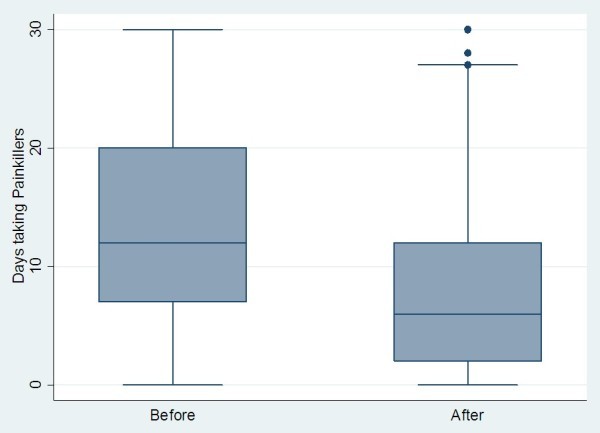
Change in days taking painkillers pre- and post-BOTOX in chronic migraine sufferers + .

A comparison of all pre- and post-treatment outcomes is shown in Table 1. As the outcomes were skewed in their distribution, the median and inter-quartile ranges (IQR) were used to summarise the responses at each time-point. The median change over time, corresponding confidence interval (CI) and p-values are also reported.Graphical illustrations of key pre- and post-treatment results are shown in Figures 2, 3, 4 and 5.

**Table 1:** Change in outcomes pre- to post-treatment

**Outcome**	**n**	**Pre-treatment Median (IQR)**	**Post-treatment Median (IQR)**	**Change Median (95% CI)**	**p-value**
Headache days	254	27 (22, 30)	18 (10, 25)	-7 (-8, -5)	<0.001
Migraine days	254	15 (10, 19)	7 (3, 12)	-6 (-8, -5)	<0.001
Crystal clear days	254	3 (0, 8)	12 (5, 20)	7 (5, 8)	<0.001
Mild days	254	10 (7, 15)	8 (4, 13)	-1 (-2, -1)	<0.001
Painkiller days	242	12 (7 ,20)	6 (2, 12)	-3 (-4, -3)	<0.001
Triptan days	241	5 (0, 8)	2 (0, 6)	0 (-1, 0)	<0.001
Days off work	58	4 (3, 6)	1 (0, 4)	2 (3, 1)	<0.001

The analysis suggested statistically significant differences between the pre-and post- treatment measurements for all outcomes examined. Headache days, migraine days, mild days, painkiller days, triptan days and days off work were all found to be significantly reduced after compared with before treatment. For example, the median number of headache days was 27 before treatment reduced to 18 after treatment (p < 0.001). Conversely, there was a significant increase in the number of headache free days from pre- to post-treatment (3 to 12 days respectively) (p < 0.001).

The 50% or 75% reduction data (and ≥2 and ≥3-fold increase in crystal clear days) are summarised in Table 2.

**Table 2:** The number of patients who achieved a ≥50% or ≥75% reduction in outcome measures following BOTOX treatment

**Outcome**	**≥50% reduction n (%)**	**≥75% reduction n (%)**
Headache days	80/254 (32%)	36/254 (14%)
Migraine days	128/254 (50%)	58/254 (24%)
Mild days	70/254 (28%)	32/254 (13%)
Painkiller days	87/243 (36%)	47/243 (19%)
Triptan days	76/242 (31%)	36/242 (15%)
Days off work	30/58 (53%)	19/58 (29%)
	**≥2-fold increase n (%)**	**≥3-fold increase n (%)**
Crystal clear days	128/254 (50%)	79/254 (31%)

Of the cohort, 80/254 (32%) reported at least a 50% reduction in headache days, 128/254 (50%) reported at least a 50% reduction in migraine days and 128/254 (50%) reported at least an increase in headache free days twice that of baseline. Also, 66 out of 254 (26%) reported an improvement in all three parameters, 106 (42%) in at least two of the three parameters and 167 (65.7%) in at least one of the three parameters. Of the cohort, 36/254 (14%) reported at least a 75% reduction in headache days, 58/254 (24%) reported at least a 75% reduction in migraine days and 79/254 (31%) reported at least an increase in crystal clear days three times that of baseline. Also, twenty out of 254 (7.8%) reported an improvement in all three parameters, 47 (18.5%) in at least two of the three parameters and 107 (42%) in at least one of the three parameters.

### Responders as per study criteria

As per the study criteria, responders were defined as having a 50% reduction in headache or migraine days or an increment in headache free days twice that of the baseline in a 30-day period.

From this, the following responder criteria were achieved:

• 87 patients (34%) met none of the criteria

• 61 patients (24%) met only one of the three criteria

• 40 patients (16%) met two of the three criteria

• 66 patients (26%) meet all three criteria.

Using Hull criteria, the authors found nearly two thirds of patients showed a meaningful response. The reduction in headache days was 32% compared to the reduction in migraine days (50%) or an increment in headache free days twice the baseline (50%). Evaluation of migraine and headache free days were, therefore, more sensitive in assessing response than headache days.

### Change in migraine severity

There were 3,855 moderate to severe headache days pre-OnabotulinumtoxinA treatment. Post- treatment, the number of moderate to severe headache days were reduced to 2,164 (-44%).

There were 2,645 mild days pre- OnabotulinumtoxinA treatment; of these, 2,234 (-16%) remained mild after treatment. There were 1,131 crystal clear days pre- OnabotulinumtoxinA treatment and 2,502 post- OnabotulinumtoxinA treatments.

### Productivity

**Figure 6:**
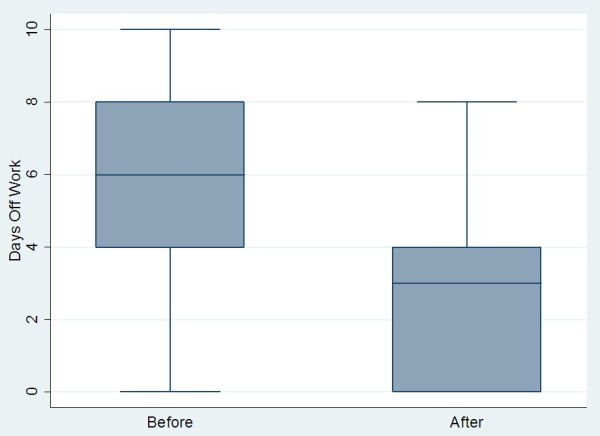
Change in days off work pre- and post-BOTOX in chronic migraine sufferers.

Data on overall days off work was available for 58/254 patients (23%); in these, the median number of days off work per month reduced from 3.5 to 1 days after OnabotulinumtoxinA (Table 1) (Figure 6). Furthermore, 53% achieved ≥50% reduction - and 29% achieved ≥75% reduction in days off work (Table 2).

### Safety and tolerability

Of the 254 patients (with all patients given the PREEMPT paradigm of 155 units injected into 31 sites), the following adverse events were observed (Table 3).

**Table 3:** Adverse events

**Adverse event observed**	**Number of patients/254 (%)**
Pain at the site of injection for at least 24 hours	38 (14.9)
Neck Stiffness	37 (14.56)
Ptosis	28 (11)
Reported but did not complain of inability to frown	15 (5.9)
Exacerbation of headache for five days	11 (4.3)
Difficulty in swallowing	5 (1.96)
Fainting during injection	3 (1.2)

### Impact on quality of life

**Figure 7:**
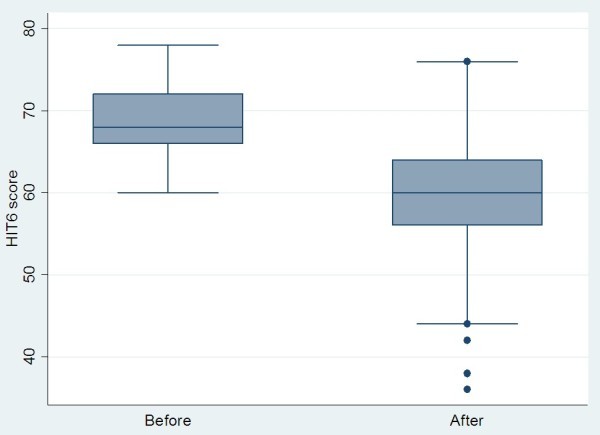
Change in HIT-6 score before and after treatment with BOTOX.

Full HIT-6 scores were available for 177/254 patients (69.9%) in the cohort. The mean and standard deviation score at each time point is shown in Table 4, along with the mean change over time, the corresponding confidence interval (CI) and the p-value. There was a mean reduction of almost 10 units in the HIT-6 score from pre- to post-treatment (p < 0.001). A graphical illustration of the before and after treatment scores is shown in Figure 7.

**Table 4:** HIT-6 score before and after treatment with BOTOX

**Outcome**	**n**	**Before treatment mean (SD)**	**After treatment mean (SD)**	**Change mean (95% CI)**	**p-value**
HIT6 score	177	68.9 (4.3)	59.2 (8.2)	-9.7 (-11.0, -8.4)	<0.001

## Discussion

This prospective analysis has shown that, in a real-life clinical setting, OnabotulinumtoxinA can effectively reduce headache days and migraine days by at least 50%, and increase headache free days from baseline in chronic migraine sufferers. OnabotulinumtoxinA use also resulted in increased work productivity. The percentage of patients who achieved at least a 50% reduction in headache days and migraine days were 32% and 50% respectively; the percentage of patients who achieved at least a 75% reduction in headache days and migraine days were 14% and 24% respectively. Furthermore, 50% of patients achieved at least a 50% increment in headache free days twice that of the baseline in a 30-day period, and 31% achieved at least a 75% increment in crystal clear days three times the baseline in a 30-day period.

This analysis introduces the Hull criteria for responders as a tool to evaluate response to OnabotulinumtoxinA. It includes headache days, migraine days and headache free days due to the importance of considering severity of headache as well as frequency. The authors noticed that patients with mild headache days often reported as headache free unless they were prompted with the term ‘crystal clear’. We propose to use the term ‘crystal clear’ in establishing true headache free days. Our data showed a reduction in headache days (32%) less than migraine days (50%) or increment in headache free days twice the baseline (50%). NICE guidance [32] used a 30% reduction in headache days as its only criteria to define a meaningful response to OnabotulinumtoxinA. However, from extensive experience, the authors believe that evaluation of headache severity through migraine days is a more valuable measure of efficacy in clinical practice. The authors propose that NICE revisits its definition of a responder. However, applying the NICE criteria of 30% reduction (rather than 50% used in the Hull criteria), the responder rate for headache days in this analysis increased from 32% to 46.5%.

Our study provides the first large prospective data on patients treated with OnabotulinumtoxinA in a real life clinical setting since the publication of PREEMPT. The PREEMPT 56-week clinical trial programme was the largest clinical programme investigating the use of OnabotulinumtoxinA as a prophylactic treatment for chronic migraine using a defined set of diagnostic criteria and defined clinically relevant outcome measures. The pooled analysis of the entire 56-week PREEMPT clinical programme supports the safety and efficacy of OnabotulinumtoxinA for the prophylactic treatment of chronic migraine. Statistically significant reductions were observed for OnabotulinumtoxinA vs. placebo for the primary efficacy variable of headache day frequency at week 56, as well as change from baseline in mean migraine days, moderate/severe headache days, and total cumulative hours of headache on headache days. Furthermore, there were statistically significant reductions in the frequency of acute headache medication use at week 56; also, of triptan intake favouring OnabotulinumtoxinA versus placebo at week 24 and statistically significant improvements from baseline at week 56 [18].

Our data supports the results and outcome from PREEMPT, although in some aspects our population was different to PREEMPT patients. In our study, 94.4% of patients had received three or more preventative treatments prior to OnabotulinumtoxinA. In the PREEMPT study, 35% of patients failed three oral therapies and 65% failed one oral therapy, suggesting a more severely affected population in our cohort. Furthermore, the number of headache days before receiving treatment was higher in this analysis [27] compared with the PREEMPT study (19.9 days in OnabotulinumtoxinA group in pooled analysis) [25], also suggesting a more severely affected population. However, only 50% of patients in this cohort fulfilled the criteria for medication overuse compared to 67% in the PREEMPT study. OnabotulinumtoxinA related adverse events were extremely low in this analysis, and no newly emerging safety signals were noted, although pain at the site of injection and neck stiffness was reported in significantly more patients than in the PREEMPT. The relatively low rate of adverse events is consistent with known tolerability profile of OnabotulinumtoxinA, and with results from the PREEMPT study.

Although this analysis is only subjective according to patient diaries, the HIT-6 results suggest an improved quality of life for chronic migraine patients using OnabotulinumtoxinA who often suffer pain, disability and anxiety from their symptoms. In addition, the improved productivity (assessed by reduced days off work) further supports this suggestion.

Patients with chronic migraine represent a treatment challenge [1,17,23], and are an important clinical, social and financial burden [4,12,13,36-38]. One analysis showed that, although the direct costs of migraine are high, 70-90% of the total cost of migraine is generally as a result of indirect costs [39]. Oral therapies traditionally used in chronic migraine are associated with limitations, e.g. lack of evidence base to support their use, adverse events and contraindications. Apart from OnabotulinumtoxinA, only topiramate (licensed for both episodic and chronic migraine) is supported by randomised, double-blind, placebo-controlled trial data [15,16]. From the authors’ clinical experience seeing hundreds of patients with chronic migraine in the specialist clinic, the authors feel that OnabotulinumtoxinA should be given after first-line treatments (e.g. tricyclic antidepressants, beta-blockers and topiramate) have been tried and failed, and before some other preventive treatments, such as sodium valproate, methysergide, and greater occipital nerve block/nerve stimulation. The authors feel that the responder rate observed in PREEMPT and this analysis justifies this position for OnabotulinumtoxinA in the care pathway. OnabotulinumtoxinA may well be preferred as a first choice prophylaxis in chronic migraine although authors feel its cost may well hinder its use as a first line.

In terms of how representative the patient cohort in this analysis is to clinical practice, from the authors’ experience, it is felt that this cohort is representative of patients seen in an average tertiary headache centre. For this reason, it can be projected that clinicians in other centres could observe similar benefits from using OnabotulinumtoxinA in their chronic migraine patients who fail oral prophylactic therapies.

Concerning study limitations, a well-known effect in migraine studies is the high placebo response rate. Furthermore, parenteral procedures are additionally associated with increased placebo response rates [40]. Clearly, this cannot be assessed in this analysis. Furthermore, the absence of an active comparator precludes comparison of the efficacy of OnabotulinumtoxinA with other therapies. However, patients included in this analysis had failed other traditional treatment options (at least one), were suffering from a considerable number of headache and migraine days and were heavily overusing acute pain medications. All of these measures improved with OnabotulinumtoxinA.

The authors do not have a comparison between those who tried one versus two versus three or more preventive treatments prior to OnabotulinumtoxinA but such an analysis will be performed as these data will become available. The authors also do not have data as to whether there is correlation between the number of headache days prior to treatment and response to OnabotulinumtoxinA. There is no doubt that patient expectations play an important role in determining whether a given treatment is effective. However, in the authors’ experience, no difference was observed between those who were treated in Allergan sponsored workshops versus those who were given treatment on the NHS.

The long-term outcome of patients treated with OnabotulinumtoxinA remains unclear. The only data available is from Rothrock et al., where 68% of patients continued to receive treatment after two years, although these patients were receiving treatment through insurance reimbursement and criteria for the continuation of treatment remains unclear [31]. The authors intend to see the outcome in this patient cohort where treatment is largely funded through the NHS based on NICE guidance where treatment must stop once the migraine becomes episodic.

### Implications in the United Kingdom (UK)

The healthcare system (NHS) in the UK offers free treatment at the point of entry. However, expensive treatments such as OnabotulinumtoxinA are subject to approval by the NICE who evaluates the cost effectiveness and the gain in Quality Adjusted Life Year (QALY) of a treatment before recommending it. Patients with chronic migraine often suffer for many years and either stays in the healthcare system or return periodically. In the current health care environment, costs of treatment as well as costs of a particular condition (direct and indirect) must, of course, be an important consideration. The UK’s NICE - now a globally considered monitor of cost-effectiveness - approved the use of OnabotulinumtoxinA in chronic migraine in 2012, indicating that it is considered to be a cost-effective option in eligible chronic migraine sufferers [33]. It can be calculated that, overall, relatively few patients with chronic migraine would be eligible for OnabotulinumtoxinA. Such a calculation needs to take into account the total adult population, the estimated prevalence of chronic migraine (1.8%) [3], and the fact that only around 20% of patients with chronic migraine receive a formal diagnosis [6]. Of sufferers, those who have failed three preventive treatments would be around one third of this number [26-28] and around 50% of chronic migraine patients respond to OnabotulinumtoxinA treatment [26,41]. Consequently, the budget impact will be small, particularly compared with the cost of wasted medications, repeat consultations, hospitalisations and the use of greater occipital nerve block or occipital nerve stimulation in these patients.

The significant improvement in work productivity with OnabotulinumtoxinA must also be considered an additional important finding for commissioners of health. Costs lost to reduced work productivity from chronic migraine are considerable, with 50% of chronic migraine sufferers losing ≥2 hours/week in the previous two weeks of their total productive time in one study [42]. Furthermore, over a three month period, chronic migraine has been shown to significantly reduce activities of daily living, for example, ability to perform household work and participate in family activities [6]. In both the PREEMPT study and in this analysis, OnabotulinumtoxinA was shown to improve productivity.

The authors acknowledge that the data reported here are a snapshot of a group of patients, and that there is a need for a more robust study which is now far more possible due to the recent NICE approval.

## Conclusions

OnabotulinumtoxinA is a valuable addition to current treatment options in patients with chronic migraine refractory to or intolerant of traditional oral prophylactic therapies. In this prospective analysis of 254 patients, OnabotulinumtoxinA significantly reduced the number of headache and migraine days, and significantly increased the number of headache free (crystal clear) days. It also improved patient’s quality of life. While the cost of OnabotulinumtoxinA may prevent its use as a first line treatment we concur the NICE view that it should be offered to patients who do not gain benefit from three oral prophylactic agents and before less favourable oral agents (Epilim, Methysergide) and invasive options of greater occipital nerve block/occipital nerve stimulation. This will also help to avoid overuse of other analgesics, including triptans.

### Clinical implications or article highlights

In this prospective analysis of 254 patients in a real-life setting of a tertiary headache clinic, OnabotulinumtoxinA significantly reduced the number of headache and migraine days, and significantly increased the number of crystal clear days. It also improved patient’s quality of life. It is believed that these results represent data on the largest post-marketing cohort of patients treated with OnabotulinumtoxinA in the real-life clinical setting. Patients who do not gain benefit from three oral prophylactic agents should be offered OnabotulinumtoxinA before the use of costly and invasive options, such as greater occipital nerve block/occipital nerve stimulation. This will also help to avoid overuse of other analgesics, including triptans.

## Competing interests

Fayyaz Ahmed has received honorarium to deliver training workshops for Allergan paid to British Association for the study of headache (BASH) and received honorarium to attend Allergan Advisory Board meetings. Modar Khalil – None. Hassan Waseem Zafar – None. Victoria Quarshie - None.

## Authors’ contributions

Fayyaz Ahmed performed all the injections and collected the data. Victoria Quarshie, Hassan Zafar and Modar Khalil entered and maintained the data and analysed it, all authors contributed to manuscript writing and review. All authors read and approved the final manuscript.
